# Snake Venom PLA_**2**_s Inhibitors Isolated from Brazilian Plants: Synthetic and Natural Molecules

**DOI:** 10.1155/2013/153045

**Published:** 2013-09-22

**Authors:** B. M. A. Carvalho, J. D. L. Santos, B. M. Xavier, J. R. Almeida, L. M. Resende, W. Martins, S. Marcussi, S. Marangoni, R. G. Stábeli, L. A. Calderon, A. M. Soares, S. L. Da Silva, D. P. Marchi-Salvador

**Affiliations:** ^1^Chemistry, Biotechnology and Bioprocess Department, Federal University of São João Del Rei, 36420-000 Ouro Branco, MG, Brazil; ^2^Molecular Biology Department, Federal University of Paraíba, 58051-900 João Pessoa, PB, Brazil; ^3^Biochemistry Department, State University of Campinas, 13083-970 Campinas, SP, Brazil; ^4^Chemistry Department, Federal University of Lavras, 37200-000 Lavras, MG, Brazil; ^5^Oswaldo Cruz Foundation, Federal University of Rondonia, 76812-245 Porto Velho, RO, Brazil

## Abstract

Ophidian envenomation is an important health problem in Brazil and other South American countries. In folk medicine, especially in developing countries, several vegetal species are employed for the treatment of snakebites in communities that lack prompt access to serum therapy. However, the identification and characterization of the effects of several new plants or their isolated compounds, which are able to inhibit the activities of snake venom, are extremely important and such studies are imperative. Snake venom contains several organic and inorganic compounds; phospholipases A_2_ (PLA_2_s) are one of the principal toxic components of venom. PLA_2_s display a wide variety of pharmacological activities, such as neurotoxicity, myotoxicity, cardiotoxicity, anticoagulant, hemorrhagic, and edema-inducing effects. PLA_2_ inhibition is of pharmacological and therapeutic interests as these enzymes are involved in several inflammatory diseases. This review describes the results of several studies of plant extracts and their isolated active principles, when used against crude snake venoms or their toxic fractions. Isolated inhibitors, such as steroids, terpenoids, and phenolic compounds, are able to inhibit PLA_2_s from different snake venoms. The design of specific inhibitors of PLA_2_s might help in the development of new pharmaceutical drugs, more specific antivenom, or even as alternative approaches for treating snakebites.

## 1. Introduction

Venomous snakebites represent an important risk for public health worldwide, especially in tropical regions where these accidents are more common. Snake venom is composed by a mixture of inorganic ions (calcium potassium, iron, cobalt, copper, and magnesium), organic compounds like carbohydrate, serotonin, histamine, bradykinin potentiating peptide, disintegrins, and proteins with or without catalytic activity (L-amino acid oxidases, lectins, hyaluronidases, serine proteases, metalloproteases, and phospholipases A_2_) [1]. The phospholipase A_2_ enzymes (PLA_2_s, E.C. 3.1.1.4, and phosphatide sn-2 acylhydrolases) are one of the most important enzymes for its effect. The PLA_2_ class includes several polypeptides with similar enzymatic functions; however, these proteins exert a variety of relevant toxic actions, such as neurotoxicity and myotoxicity [2].

Secreted phospholipases A_2_ (sPLA_2_s) catalyze the hydrolysis of glycerophospholipids in sn-2 position and promote the release of lysophospholipids and fatty acids, such as the arachidonic acid. The arachidonic acid is a precursor of prostaglandins and leukotrienes, and it is involved in inflammatory process characterized by increase by microvascular permeability and oedema formation, leukocyte recruitment into tissues, nociception, and release of inflammatory mediators which mimic a number of systemic and local inflammatory disorders in humans [1–5]. In addition, the excess levels of sPLA_2_s were associated with many physiopathological processes as cerebral illnesses, cardiovascular disorders, cancers, asthma, respiratory distress syndrome, and progression of tonsillitis [4–8]. On the other hand, the increased sPLA_2_ activity is observed in some brain tumours, in chronic neurological disorders associated with neurodegenerative diseases, such as neural trauma, Alzheimer's, and Parkinson's diseases, and may serve as a marker of increases in permeability of the blood-cerebrospinal fluid barrier [9, 10].

PLA_2_s show considerable identity in their amino acid sequence [11, 12], but the three-dimensional structure similarity among group II sPLA2s is considerably higher, and this fact shows the importance of the 3D structure for the biological activities [2, 13–16]. Venom of different snake species is used as sources of PLA_2_, due to the abundance of these enzymes and the fact that the purification of these molecules is relatively simple [17–19].

The apparent contradiction between structural uniformity and functional diversity, exhibited by PLA_2_s, has attracted much interest from the scientific community. According to Ohno and collaborators [20], this diversity of pharmacological and toxic effects may have been evolutionarily acquired by positive Darwinian selection of the coding exons of these activities.

Due to a high degree of structural similarity between the sPLA_2_s from snake venom and the human, it is a prerequisite to use the snake venom PLA_2_ inhibitors for the design of new drugs for human diseases because the new inhibitory drugs must be related to the transitional state of the enzyme [2, 21]. Small variations among PLA_2_ isoforms may be used for the study of structural and functional relationships of these proteins. Moreover, research regarding natural and synthetic inhibitors that are able to neutralize the toxic effects promoted by these enzymes is being carried out in an attempt to explain the physiopathological mechanisms of these molecules [22–24]. Furthermore, knowledge about the mechanism of toxicity exhibited by these proteins may assist the discovery and development of new anti-inflammatory drugs, cellular lesions, and therapies for several diseases, including Parkinson's, Alzheimer's, and even cancer [12, 25–29].

Treatment of snakebites is still carried out using traditional antivenom therapy [30]. However, although antivenom therapy is effective for the majority of cases, some side effects exist for these treatments, including adverse reactions on the skin, gastrointestinal tract, and respiratory and circulatory systems [31, 32]. Moreover, snake antivenom therapy is usually unable to prevent the progress of local effects [30]. Given the limitations of traditional therapy, research focusing on the interactions between PLA_2_s and their natural or synthetic inhibitors could allow the development of alternative treatments for the toxic and pharmacological effects of snake bites [23, 33]. Plant extracts have become a promising alternative to substitute traditional snake antivenom, which often are unavailable in emergency situations [34, 35]. After studying plants commonly used to treat snakebites in South America, Soares and collaborators [34] reported 56 vegetal species that exhibited anti-inflammatory activity caused by crude snake venom or by their isolated components.

## 2. PLA_2_ Inhibitors Isolated from Plants

Plants are used in traditional medicine to treat the effects of venomous snake bites. Pharmacological studies have shown that fractions of these plant extracts have anti-inflammatory, antiviral, and antivenom properties [36, 37]. The effect of specific molecules from these plant extracts may be attributed to the presence of multiple factors, such as low molecular weight of chemical compounds and the abundance of chemical and pharmacological properties [33].

Borges et al. [38] reported that the aqueous extract of *Casearia sylvestris *(Flacourtiaceae), a native vegetal species found in Brazilian open pastures, had the ability to inhibit myotoxic, anticoagulant, and edema-inducing activities from *Bothrops moojeni*, *B. pirajai*, *B. neuwiedi,* and *B. jararacussu* venom and its Asp49 and Lys49-PLA_2_ isolated toxins. In addition, Borges and collaborators [39] emphasized that *C. sylvestris* was able to neutralize hemorrhagic activity caused by the *B. pirajai*, *B. jararacussu*, *B. asper*, *B. moojeni,* and *B. neuwiedi* venom. Cavalcante and collaborators [22] showed that the *C. sylvestris* aqueous extract demonstrated protective effects against muscle damage induced by two Lys49-PLA_2_ toxins (PrTX-I from *B. pirajai* and BthTX-I from *B. jararacussu* snake venom) and prevented the neuromuscular blockage induced by all PLA_2_ toxins.

*Mandevilla velutina *(Apocynaceae) is a perennial plant from the Brazilian *cerrado* that has been studied for its anti-inflammatory activity, as well as its antagonist effect on bradykinin, a vasodilator [40]. These authors reported that the aqueous extract of this plant was an effective inhibitor of phospholipase A_2_ activity and some toxic effects, such as hemorrhage, caused by venom from snakes of the *Bothrops* and *Crotalus* genus. In a posterior study, the same research group reported that extracts from *Mandevilla illustris* were able to completely inhibit the activity of the Crotoxin B, the basic Asp49-PLA_2_, isolated from *Crotalus durissus terrificus *venom [41].

The antihemorrhagic properties of the aqueous extract of *Pentaclethra macroloba* (Fabaceae), an ethnomedicinal plant found in the Amazon region, were evaluated against snake venom and displayed a full inhibition of hemorrhagic and nucleolytic activities induced by several snake venom. Additionally, a partial inhibition of myotoxic, lethal, enzymatic and edema activities of snake venom, and their isolated PLA_2_s was observed [42].

Almeida and collaborators [43] showed that the aqueous extract of *Tabernaemontana catharinensis* (Apocynaceae), which is encountered in some countries of South America, was able to inhibit the crotoxin complex, isolated from *C. d. terrificus* venom, and was also able to partially neutralize the myotoxicity of *B. jararacussu* snake venom and its basic PLA_2_s [44].

The aqueous extract of the aerial parts of *Bauhinia forficate* (Fabaceae), a species native to Asia and well adapted and developed in several regions of Brazil, was assayed against the fibrinogenolytic and coagulant activities of *C. d. terrificus* and *B. jararacussu *crude venom and was found to neutralize these effects. Moreover, the extract efficiently inhibited the edema induced by *C. d. terrificus* venom and its isolated PLA_2_ [45].

Mendes and collaborators [46] reported that the aqueous extract of *Schizolobium parahyba* (Fabaceae), a plant found in the Mata Atlântica of southeastern Brazil, contains compounds that can inhibit some enzymatic and biological activities induced by *Bothrops pauloensis* (current *Bothropoides paulensis*) and *C. d. terrificus* snake venom as well as by their isolated neuwiedase toxins (metalloproteinase), BnSP-7 (basic Lys49-PLA_2_ from *B. paulensis* venom), and Crotoxin B.

The ethanolic extract of the aerial parts of *Blutaparon portulacoides* (Amaranthaceae), an herbaceous plant that occurs mainly in the Atlantic bush, caused a reduction in edema formation and in the leukocyte influx induced by Lys49-PLA_2_ and isolated from *B. jararacussu* venom [47].

In 2005, Maiorano and collaborators [48] evaluated aqueous extracts prepared from dried or fresh roots, stems, or leaves of *Mikania glomerata* (Asteraceae), a plant found in the Mata Atlântica in Southeastern Brazil and popularly known as “Guaco.” The *M. glomerata* extract efficiently neutralized different toxic, pharmacological, and enzymatic effects induced by *Bothrops* and *Crotalus* snake venom. The phospholipase A_2_ activity and the edema induced by *C. d. terrificus* venom were inhibited by approximately 100 and 40%, respectively, and this inhibition was also partially observed with the *Bothrops* venom. The hemorrhagic activities of *B. alternatus*, *B. moojeni*, *B. neuwiedi*, and *B. jararacussu* venom were significantly inhibited by *M. glomerata* extract, while the clotting activities of *C. d. terrificus*, *B. jararacussu*, and *B. neuwiedi* venom were totally inhibited. In addition, Floriano and collaborators [49] reported clinical and laboratory alterations in mice caused by the combination of *M. glomerata* leaf extract and antiophidian serum against *C. d. terrificus* venom.

Nazato and collaborators [50] found that the methanolic extract of the bark from *Dipteryx alata* (Fabaceae) (a native species of the Brazilian Savanna, found principally in Minas Gerais, Goiás, Federal District, and Mato Grosso) decreased the neurotoxicity and myotoxicity of *B. jararacussu* crude venom. In another study by Puebla and collaborators [51], the extract from *D. alata* was fractionated and its compounds were evaluated against the neuromuscular blockade caused by *B. jararacussu* venom.

The ability of the ethanolic extract of *Hypericum brasiliense* (Hypericaceae), a plant found mainly in the southeastern and southern regions of Brazil, to neutralize some effects induced by *B. jararaca *venom was investigated using biological assays. *H. brasiliense* extracts were able to inhibit some pharmacological effects such as lethality, edema, hemorrhage, hemolysis and, proteolysis, as well as fibrinogen or plasma clotting [52].

In 2012, Dey and De [53] published a review that evaluated several pharmacological studies on plant efficacies against snakebites. The authors compiled studies from a number of plants or their fractionsthat were active against snake venom and concluded that folk knowledge is relevant. However, clinical tests should be performed with these plant extracts or fractions to assess the effect of the compounds used for the treatment of snakebites.

Recently, Samy et al. [54] published an extensive revision on the therapeutic application of natural inhibitors of snake venom PLA_2_s, covering molecules from the primary metabolism of different organisms, such as glycoproteins (PLIs), peptides, and lipids, as well as from secondary metabolism, exemplified by terpenoids, alkaloids, flavonoids, and other molecules. These authors concluded that the biotechnological potential of PLA_2_ inhibitors may provide therapeutic molecular models with antiophidian activity to supplement conventional serum therapy or for the development of novel antivenom therapeutics. Additionally, inhibitors isolated from medicinal plants may also be an essential tool in isolated communities [23, 54].

## 3. Structural Characterization of PLA_2_ Inhibitors

The main classes of PLA_2_ inhibitors are the phenolic compounds, which include flavonoids, coumestans and alkaloids, steroids and terpenoids (mono-, di-, and triterpenes), and polyphenols (vegetable tannins). There is also mention in the literature of other molecules such as carbohydrates, lipids, and proteins, although this paper emphasizes molecules originating from plant secondary metabolism.

### 3.1. Phenolic Compounds

#### 3.1.1. Flavonoids

**Figure 1:**
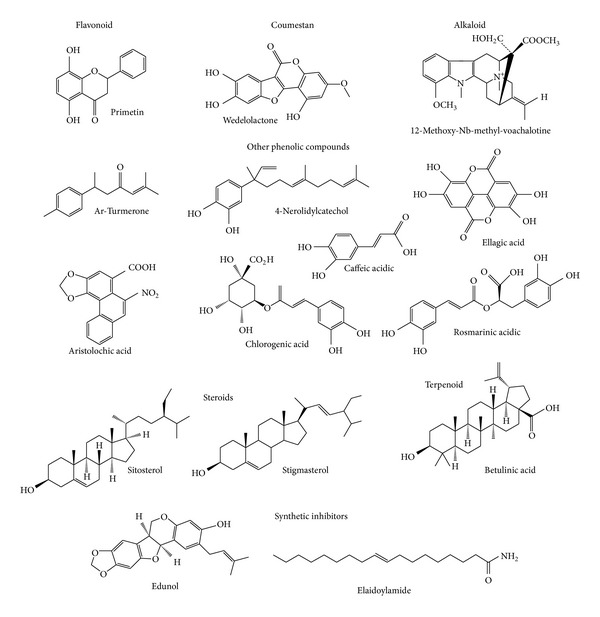
Structures of bioactive compounds with inhibitory potential against the snake venom or its phospholipase A_2_ fraction. Draw using ACD/ChemSketch program (http://www.acdlabs.com/).

Polyphenolic secondary metabolites are commonly able to bind to biological polymers, and some of these have been shown to inhibit PLA_2_s. Examples include quercetin, a strong lipoxygenase inhibitor, naringenin, artemetin, kaempferol, and galangin, among several other flavonoids. Primetin (Figure 1), 5,8-dihydroxyflavone, isolated from *Primula *sp. (Primulaceae), is known for its ability to inhibit toxins from snake venom; its structural form may be seen in Figure 1. Flavonoids usually exert their inhibitory effect via hydrophobic interactions with the A and B rings and aromatic or hydrophobic amino acid residues in the protein [36, 55, 56].

#### 3.1.2. Coumestans

*Eclipta alba* (Asteraceae) is a native plant from Brazil and other tropical and subtropical areas of the world whose medicinal properties are widely known. *E. alba* was genetically engineered using *Agrobacterium rhizogenes *LB9402 to enhance the production of secondary wedelolactone metabolites, which are coumestan compounds with activity against basic PLA_2_s. This mutant strain was found to reduce the phospholipase A_2_ activities and myotoxic and neurotoxic effects of the *C. d. terrificus* and *B. jararacussu* snake venom [37, 57]. Analogs of wedelolactone molecule (Figure 1) were able to antagonize the release of creatine kinase induced by *B. jararacussu* venom even at concentrations as low as 30 *μ*M [58, 59].

#### 3.1.3. Alkaloids

Batina and collaborators [60] isolated an alkaloid from *Tabernaemontana catharinensis *(Apocynaceae) named 12-methoxy-4-methylvoachalotine (Figure 1) and reported a strong inhibitory effect against lethality and myotoxic activities induced by *C. d. terrificus* venom.

#### 3.1.4. Other Phenolic Compounds

Ar-Turmerone (Figure 1) is a phenolic compound isolated from the *Curcuma longa* (Zingiberaceae) plant that has a strong effect against the hemorrhage and lethality caused by *B. jararaca *and *C. d. terrificus* snake venom [61].

Extracts from *Piper umbellatum *and* P. peltatum *(Piperaceae)were shown to inhibit the myotoxic activities of PLA_2_s isolated from *Bothrops* snake venom [62]. Fractionation of these plant extracts revealed that 4-nerolidylcatechol, a hydroxylated phenolic compound (Figure 1), was responsible for at least part of the inhibitory effect against groups I, II, and III of PLA_2_s.

In 2008, Da Silva and collaborators [63] studied the half maximal inhibitory concentration (IC_50_) of ellagic acid (Figure 1), extracted from *C. sylvestris, *against BthTX-II, a basic Asp49-PLA_2_ from *B. jararacussu* snake venom, and concluded that this compound was effective at competitively inhibiting the induction of edema, myotoxicity, and enzymatic activities, incurred by this PLA_2_.

The first structural analysis of aristolochic acid (Figure 1), isolated from *Aristolochia *sp. (Aristolochiaceae), was performed by Vishwanath and Gowda [64]. In this study, the interaction of aristolochic acid, an alkaloid, with PLA_2_ from *Vipera russelli* was characterized as noncompetitive inhibitive. This compound has also been shown to reduce the induction of edema by this enzyme. Additionally, Vishwanath et al. [65] emphasized that the interaction between aristolochic acid, from *Aristolochia radix,* and three PLA_2_s from *Trimeresurus flavoviridis* resulted in the inhibition of hemolytic and edema induction by competitive inhibition. Chandra and collaborators [66] reported the crystal structure of the complex formed between the PLA_2_ isolated from *V. russelli* venom and aristolochic acid. In this study, the interaction between aristolochic acid and PLA_2_ was competitive, and the final model consists of a dimer of PLA_2_ and one molecule of aristolochic acid located in the binding site of molecule A; this interaction was stabilized by three hydrogen bonds and hydrophobic contacts.

Chlorogenic and caffeic acids (Figure 1) can interact with proteins via hydrophobic contacts and hydrogen bonds, inhibiting enzyme function and acting as antidotes. Strong interactions may induce conformational changes in the protein structure [26]. In 2011, Shimabuku and collaborators [67] crystallized PrTX-I (basic Lys49-PLA_2_ from *B. pirajai *snake venom) in the presence of the inhibitor, caffeic acid, and the electron-density map which unambiguously indicated the presence of three caffeic acid molecules interacting with the C-terminus of the protein.

Rosmarinic acid (Figure 1) is a hydroxylated phenolic compound isolated from *Cordia verbenacea* (Boraginaceae). This compound demonstrates antimyotoxic properties and inhibits edema induced by crude *B. jararacussu* snake venom and its basic PLA_2_s [36, 55]. The three-dimensional structure of the PrTX-I, rosmarinic acid complex, was elucidated by Santos and collaborators [68], where rosmarinic acid was observed located at the entrance of the hydrophobic channel monomer A of the PrTX-I dimer via an interaction between hydrogen bonds and hydrophobic contacts in the same monomer. Interactions were also observed between rosmarinic acid and a residue of the C-terminal region of the monomer B. The interaction between the rosmarinic acid molecule with the hydrophobic channel (monomer A) and the C-terminal region (myotoxic site, monomer B) suggests two mechanisms of myotoxicity inhibition [68].

### 3.2. Steroid Compounds

Sterol and cholesterol molecules present well-known antidote activities against snake venom. Steroids can form complexes that are stabilized via Van der Waals interactions, as well as by hydrophobic interactions [37]. Antimyotoxic and antihemorrhagic effects of the *Eclipta prostrata* (Asteraceae) extract and its components, sitosterol and stigmasterol (Figure 1), were observed against *B. jararaca*, *B. jararacussu*, and *Lachesis muta* snake venom [37, 69]. Previously, Mors [70] reported that sitosterol and stigmasterol, isolated from *E. prostrata,* prevented the lethality of the *C. d. terrificus* venom in a dose-dependent manner.

### 3.3. Terpenoids

The neoclerodane, diterpenoid, isolated from the aerial parts of* Baccharis trimera* (Asteraceae), demonstrate anti-hemorrhagic and antiprotolithic properties against *Bothrops* snake venom [71] 

Several pentacyclic triterpenes, such as oleanolic acid, lupeol, ursolic acid, taraxerol, taraxasterol, *α*,*β*-amyrin, and friedeline, exhibit activity against snake venom [37]. Triterpenoids, isolated from *Betula alba* (Betulaceae), including pentacyclic triterpenes betulin and betulinic acid (Figure 1), exhibited antiphospholipasic A_2_ activity. Docking (*in silico* experiments) indicated betulinic acid as the best PLA_2_ inhibitor, due to its direct insertion in the catalytic site on the enzyme, with a very low energy value [55].

### 3.4. Synthetic Inhibitors

Edunol (Figure 1) is a pterocarpan with a chemical structure similar to those of the inhibitors extracted from the roots of *Harpalyce brasiliana* (Fabaceae). Edunol was obtained via chemical synthesis, and the compound showed anti-myotoxic, anti-proteolytic, and anti-PLA_2_ activities against *B. jararacussu* crude venom [55, 72].

Elaidoylamide, the amide of trans-9-octadecenoic acid (Figure 1), is a powerful synthetic inhibitor of a neurotoxic Asp49-PLA_2_ from *Vipera ammodytes meridionalis* venom. In 2003, Georgieva and collaborators [73] isolated the neurotoxic complex from *V. a. meridionalis* venom, dissociated the basic PLA_2_ from the complex, and crystallized it with elaidoylamide. This final structure contained two identical homodimers and one molecule of elaidoylamide bound simultaneously to the substrate-binding sites of each homodimer [74].

Villar and collaborators [33] demonstrated that synthetic inhibitor derivatives from nitrostyrene that contain typical nitro groups at the *ortho-*, *meta-*, and *para-* positions on the aromatic ring were more efficient against the enzymatic, edematogenic, and myotoxic activities of PLA_2_s from *B. jararacussu* venom. Da Silva and collaborators [75, 76], performing molecular modeling studies between Asp49-PLA_2_ from *C. adamanteus* venom and synthetic derivatives polyhydroxy phenolic compounds, concluded that some conformations of these groups might positively influence enzymatic activity inhibition.

Isolated inhibitors (natural or synthetic) can be important tools for understanding the mechanisms of action of PLA_2_s from snake venom, and, consequently, these results might be helpful for the design of a drug that specifically inhibits PLA_2_s. However, the synthesis of compounds analogous to their natural equivalents, based on chemical characteristics or with minor structural modifications, is often necessary. The synthesis of compounds could be justified by the low amount of these compounds available in vegetal extracts or to adjust some specific chemical characteristics. For this reason, some researchers have isolated and characterized new compounds or produced synthetic analogues for use in the commercial production of pharmaceutical drugs.
